# Lung Function Improvement in Bronchial Asthma: A Study of Sublingual Immunotherapy

**DOI:** 10.1002/pdi3.70022

**Published:** 2025-09-28

**Authors:** Yinming Song, Daiyin Tian, Sha Liu, Fangjun Liu, Jingyue Liu, Ying Li, Li Yan

**Affiliations:** ^1^ National Clinical Research Center for Child Health and Disorders Ministry of Education Key Laboratory of Child Development and Disorders Chongqing Key Laboratory of Pediatric Metabolism and Inflammatory Diseases Department of Respiratory, Children's Hospital of Chongqing Medical University Chongqing China

**Keywords:** airway hyperresponsiveness, bronchial asthma, dust mites, lung function, sublingual immunotherapy

## Abstract

This is a retrospective study. In order to investigate the effect of specific immunotherapy on the improvement of lung function and symptoms in children with bronchial asthma, 256 children with bronchial asthma were selected and divided into an experimental group and a control group according to whether they chose to use sublingual specific immunotherapy or not. The control group was given basic drug therapy, and the experimental group was given specific immunotherapy on the basis of basic drug therapy. Differences between the two groups were compared in terms of lung function, efficacy, and asthma medication dose. The results showed that peak expiratory flow (PEF), maximum expiratory flow at 25% of forced vital capacity (MEF_25_), maximal mid‐expiratory flow curve (MMEF), maximum expiratory flow at 75% of forced vital capacity (MEF_75_), maximum expiratory flow at 50% of forced vital capacity (MEF_50_), and airway hyperresponsiveness improved in both groups after 1 year of treatment (*P* < 0.05), and the result of experimental group was better than that of the control group (*P* < 0.05). This study shows that sublingual specific immunotherapy combined with inhaled corticosteroid therapy has positive therapeutic effects on asthma patients, which can reduce the dose of medication used in asthma patients and improve symptoms.

## Graphical abstract

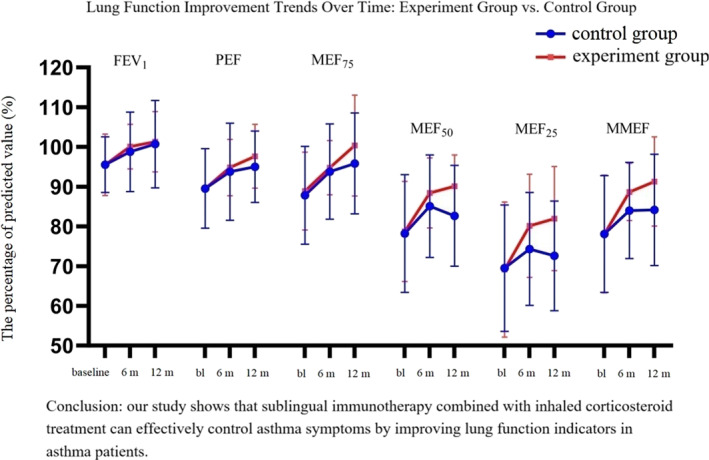

## 1 Introduction

Asthma is a major public health problem with clear socioeconomic implications. Current conventional treatments for asthma are inhaled and oral medications, which effectively control symptoms and the ongoing inflammatory process, but do not affect the underlying, dysregulated immune response. Thus they are very limited in controlling disease progression. In contrast, allergen immunotherapy (AIT) is the only known treatment that alters the natural course of allergic disease and induces long‐term remission, that is, it has a continuity of action and is able to remain efficacious beyond the completion of the course of treatment. Studies have shown that AIT builds and maintains long‐term tolerance to allergens and therefore improves symptoms and reduces the need for medication, thereby improving the quality of patient survival and reducing financial stress on families [1]. Sublingual immunotherapy (SLIT) has the advantages of significant efficacy, convenient application, high safety, and controllability, and has been widely used in China, Europe, Japan, and other countries and regions. However, studies related to the improvement of lung function and efficacy of SLIT in pediatric asthma patients have not yet reached a clear conclusion, so this study aimed to investigate the effects of SLIT, lung function, efficacy, and on the dose of asthma medication.

## 2 Materials and Methods

### 2.1 Patients

A total of 256 patients with bronchial asthma who were initially diagnosed in the respiratory specialty outpatient clinic of Children's Hospital of Chongqing Medical University and underwent 1‐year follow‐up treatment from January 2020 to June 2021, were selected and divided into a test group and a control group based on voluntary choice of whether to use sublingual specific immunotherapy. The experimental group was treated with sublingual specific immunotherapy combined with inhaled corticosteroids (ICS) therapy, with a total of 114 patients, including 61 males and 53 females, aged 4.01–12.87 years, with a mean age of (6. 92 ± 2.26) years. The control group was treated with single ICS therapy, with a total of 142 patients, including 73 males and 69 females, aged 4.01–12.93 years, and a mean age of (6.92 ± 2.06) years. There was no statistical difference in the basic data of the two groups of patients, and they were comparable (*P* > 0.05) (Table 1). This study was reviewed by the Medical Ethics Committee of Children's Hospital of Chongqing Medical University (approval number: 2023, 505).

**TABLE 1:** Baseline characteristics.

Group	Age (year, *X* ± *S*)	Sex (male, %)	Airway hyperresponsiveness (n, %)	Initial inhaled corticosteroid dosage (ug)
Experiment group	6.92 ± 2.26	0.54	112 (98.2)	250 (200, 250)
Control group	6.92 ± 2.06	0.51	141 (99.3)	250 (200, 250)
*p*	> 0.05	> 0.05	> 0.05	> 0.05

Inclusion criteria: all of them met the diagnostic criteria of bronchial asthma in the Guidelines for the Diagnosis and Prevention of Bronchial Asthma in Children [2]: (1) there were recurrent episodes of wheezing, shortness of breath, cough and chest tightness, and most of them were related to exposure to allergens, cold air, physical and chemical stimuli, and respiratory infections, with more frequent occurrences in the nighttime and early morning hours; (2) diffuse or scattered rumbling sounds and prolonged expiratory phases were audible in the episodes; (3) the above signs and symptoms are effective or resolved spontaneously after anti‐asthma treatment; (4) cough, wheezing, shortness of breath, and chest tightness caused by other diseases are excluded; and (5) those with atypical clinical manifestations need to have at least one of the following conditions: ① Confirmation of the existence of reversible airflow limitation: (a) positive bronchodilator test: ≥ 12% increase in forced expiratory volume in 1 second (FEV1) 15 min after inhalation of fast‐acting β2 agonists; (b) improvement of pulmonary improvement in ventilatory function: increase in FEV1 ≥ 12% after administration of inhaled glucocorticosteroids and anti‐leukotriene drugs for 4–8 weeks; ② positive bronchial provocation test; and ③ day‐to‐day variability of peak expiratory flow (PEF) (monitored continuously for 2 weeks) ≥ 13%. The diagnosis of asthma is made if the following conditions are met: (i) a positive FEV1; (ii) a positive bronchial provocation test; and (iii) a positive PEF day‐to‐day variability (2 weeks of continuous monitoring) ≥ 13%, and meet the following conditions: ① allergen is dust mite positive, dust mite skin prick test (SPT) or serum sIgE ≥ grade 2, combined or not combined with other allergens; ② good compliance: the patient can adhere to regular follow‐up every 2–3 months and cooperate with the lung function and related tests; and ③ 4 weeks before enrollment did not receive immunotherapy and inhaled corticosteroid therapy.

Exclusion criteria: (1) severe uncontrolled asthma (FEV1< 70%); (2) used β‐blockers or angiotensin‐converting enzyme (ACE) blockers or monoamine oxidase inhibitors; (3) comorbidities with other serious diseases, such as uncontrolled cardiovascular disease, active pulmonary tuberculosis, immune disorders, and malignancies; and (4) poor compliance, serious psychological disorders, or inability to understand the risks and limitations of treatment [3].

### 2.2 Study Protocol

All patients completed an initial pulmonary function test before enrollment; all had their level of asthma control assessed; and all completed allergen testing (SPT, suggestive of dust mite positivity [++ and above] or dust mite serum sIgE ≥ grade 2).

The control group was treated with small to moderate inhaled doses of ICS and long‐acting β2 agonists according to the control of symptoms and lung function, and was followed up regularly every 3 months for a total of 12 months. During the follow‐up, the asthma medication was adjusted according to the Global Initiative to Control Asthma (GINA) stepwise program, with a treatment cycle of 1 year [4], and the changes in pulmonary function, asthma symptom, assessment of the level of asthma control, and the dose of ICS were recorded in detail at each follow‐up visit.

ICS: (1) fluticasone propionate inhalation aerosol, trade name: Coleusinone; (2) salmeterol ticasone aerosol, trade name: Sulpiride; and (3) budesonide formoterol powder inhaler, trade name: Cytotec Dobo inhaler.

Treatment regimen of the experiment group: addition of SLIT to the treatment regimen of the control group.

Dust mite drops, trade name: Changdi, specification: No. 1 2 mL (1 μg/mL), No. 2 2 mL (10 μg/mL), No. 3 2 mL (100 μg/mL), No. 4 2 mL (333 μg/mL), and No. 5 2 mL (1000 μg/mL). How to use dust mite drops: put the dust mite drops under the tongue, hold it for 1 min, and then swallow it. The medicine is administered once daily, usually at a fixed time of the day, and the dose is adjusted according to the degree of allergy. Desensitization therapy includes a build‐up phase and a maintenance phase. For the build‐up phase, dust mite droplets No. 1, No. 2, and No. 3 are used sequentially in week 1, 2, and 3 respectively. During each of these three weeks, the droplets are administered sublingually with daily doses following the sequence: 1 drop, 2 drops, 3 drops, 4 drops, 6 drops, 8 drops, and 10 drops. For the maintenance phase, starting from Week 4, dust mite droplets No. 4 is used, with 3 drops administered each time and once a day. The total follow‐up treatment cycle is 12 months.

### 2.3 Assessment Indicators

#### 2.3.1 Lung Function

Patients were tested using a spirometer (manufactured by Jaeger, Germany), with the test conducted by a lung function specialist. FEV1, PEF, maximal mid‐expiratory flow curve (MMEF), maximum expiratory flow at 75% of forced vital capacity (MEF_75_), maximum expiratory flow at 50% of forced vital capacity (MEF_50_), and maximum expiratory flow at 25% of forced vital capacity (MEF_25_) were recorded, expressed as percentages of the predicted values. Patients were not allowed to use bronchodilators within 24 h before the test.

#### 2.3.2 Airway Hyperresponsiveness

Continuous nebulization was performed using a jet nebulizer. Before the procedure, patients' conditions were asked in detail to ensure that the operation was conducted during the non‐asthmatic phase. During the test, patients were instructed to hold the mouthpiece with their mouths and breathe calmly and evenly with tidal breathing. The mass concentrations of inhaled methacholine (MCH) were 0.5, 2, 8, and 16 mg/ml in sequence. The test was stopped when FEV1 decreased by more than 20%, obvious discomfort or clinical manifestations appeared, or the maximum concentration was reached. The cumulative provocative dose of inhaled hormone causing a more than 20% decrease in FEV1 (PD20‐FEV1) was recorded. PD20‐FEV1 was negative when it reached 16 mg/ml without obvious discomfort or clinical manifestations; otherwise, it was positive.

#### 2.3.3 ICS Dose

Inhaled corticosteroids therapy regimen for asthma patients was developed according to the Guidelines for the Diagnosis and Prevention of Bronchial Asthma in Children [2], and the treatment regimen was evaluated every 1–3 months and adjusted accordingly. If asthma is controlled and maintained for at least 3 months, the treatment regimen may be considered downgraded until the lowest dose for asthma control is achieved. And the medication may be discontinued if the condition is stabilized after 3–6 months of controlled therapy using the lowest inhaled corticosteroid dose.

#### 2.3.4 Symptom Scores

Daytime symptom score: 0 point, no symptoms; 1 point, few symptoms and short duration; 2 point, 2 or more very short symptoms; 3 point, mild symptoms for more of the day, but with little impact on work and life; 4 points, severe symptoms for more time of the day, with impact on life and work; and 5 point, symptoms so severe that they are unable to work and live normally. Nocturnal symptom score: 0 point, no symptoms; 1 point, waking up once or waking up early; 2 point, waking up twice, including early waking up; 3 point, waking up many times; and 4 point, unable to fall asleep at night [5].

#### 2.3.5 Evaluation of Efficacy

The efficacy was determined according to the asthma diagnostic criteria in the Guidelines for the Diagnosis and Prevention of Bronchial Asthma in Children [2], marked efficacy: patients' symptoms such as coughing, wheezing and chest tightness were significantly relieved or disappeared, and their lung function was significantly improved or basically restored to normal; effective: patients' occasional coughing, wheezing, chest tightness, and their lung function was improved compared with the baseline; and ineffective: patients' symptoms or lung function did not improve, or even worsened. Total effective rate = marked efficacy rate + effective rate.

#### 2.3.6 Allergen Testing

In this study, we used SPT and allergen sIgE detection. sI grading was used in the SPT, and sI was divided into 4 grades, 0.3 ≤ sI < 0.5, +; 0.5 ≤ sI < 1.0, ++; 1.0 ≤ sI < 2.0, +++; and sI ≥ 2.0, ++++. The sIgE level to mites was measured by the Western blot method in our hospital. A sIgE level < 0.35 kU/L was defined as negative, while a sIgE level ≥ 0.35 kU/L was defined as positive. The sIgE levels could be classified into 6 grades: Grade 1 was 0.35–0.70 kU/L; Grade 2 was 0.70–3.5 kU/L; Grade 3 was 3.5–17.5 kU/L; Grade 4 was 17.5–50 kU/L; Grade 5 was 50–100 kU/L; and Grade 6 was > 100 kU/L.

### 2.4 Statistical Analysis

Data were statistically analyzed using SPSS 26.0 software. All measurements were tested for normal distribution, and *t*‐test was used for the measurements that met the normal distribution. The median (lower quartile and upper quartile) was used for the measurements that did not meet the normal distribution; the Mann–Whitney test was used for the comparison between the two groups, and the Friedman test was used for the comparison between the groups at different time points. The counting data of the two groups were expressed as the number of cases, and the comparison was performed by the Chi‐squared test. Differences were considered statistically significant when *P* < 0.05.

## 3 Results

### 3.1 Comparison of Pulmonary Function Indexes Between the Two Groups

FEV1: there was no significant difference between the two groups in FEV1 baseline comparison (*P* > 0.05). After 6 months of treatment, the difference in FEV1 between the two groups was statistically significant (*P* < 0.05), and after 1 year of treatment, there was no difference in the comparison of FEV1 between the two groups (*P* > 0.05). The difference within the control group at each time point was statistically significant (*P* < 0.05), and FEV1 was significantly higher than baseline after 1 year of treatment (*P* < 0.05); and the difference in the comparison of the each time point within the experiment group was statistically significant (*P* < 0.05), and FEV1 was significantly higher than baseline after 6 months and 1 year of treatment (*P* < 0.05).

PEF: There was no significant difference in the baseline comparison of PEF between the two groups (*P* > 0.05), and the difference in the comparison of PEF between the two groups was not statistically significant after 6 months of treatment (*P* > 0.05). And there was a statistically significant difference in the comparison of PEF between the two groups after 1 year of treatment (*P* < 0.05). The difference in the comparison of the control group at all time points was statistically significant (*P* < 0.05), and PEF was significantly higher than that of the baseline after 1 year of treatment (*P* < 0.05); and the difference in the comparison of the time points in the experiment group was statistically significant (*P* < 0.05), and the PEF was significantly higher than the baseline after 6 months and 1 year of treatment (*P* < 0.05).

Small airway function indexes: there was no significant difference in the baseline comparison of small airway function indexes of MMEF, MEF_75_, MEF_50_, and MEF_25_ between the two groups (*P* > 0.05), and the difference in the comparison of small airway function indexes between the two groups was statistically significant after 6 months and 1 year of treatment (*P* < 0.05). The difference in the comparison of the control group at each time point was statistically significant (*P* < 0.05), and the small airway function indexes were significantly higher than baseline after 1 year of treatment (*P* < 0.05). The difference between the comparison of the time points in the experiment group was statistically significant (*P* < 0.05), and the MEF_75_ was significantly higher than baseline after 6 months and 1 year of treatment (*P* < 0.05). (Table 2).

**TABLE 2:** Comparison of various indicators of lung function before and after treatment in the experiment and control groups.

Test	Groups	The number of patient (*n*)	Baseline (M, [P₂₅, P₇₅])	6 months (M, [P₂₅, P₇₅])	1 year (M, [P₂₅, P₇₅])	*χ* ^2^	*p*
FEV1	Control group	142	95.50 (88.43, 103.93)	98.78 (88.70, 106.83)	100.70 (89.65, 108.05)	9.802	0.007
	Experiment group	114	95.50 (87.35, 104.23)	100.10 (94.55, 107.13)	101.30 (93.18, 108.23)	19.943	0.000
	*Z*		−0.277	−2.061	−1.529		
	*p*		0.782	0.039	0.126		
PEF	Control group	142	89.55 (79.40, 99.23)	93.75 (81.55, 101.63)	95 (85.88,103.00)	12.448	0.002
	Experiment group	114	89.55 (80.75, 103.40)	94.81 (87.73, 104.98)	97.65 (89.70, 107.90)	26.263	0.000
	*Z*		−1.114	−1.942	−2.510		
	*p*		0.265	0.052	0.012		
MEF_75_	Control group	142	87.81 (75.25, 98.50)	93.80 (81.88, 102.68)	95.85 (83.15, 104.83)	14.423	0.001
	Experiment group	114	88.90 (79.10, 102.18)	94.77 (87.95, 107.53)	100.35 (87.90, 112.25)	23.842	0.000
	*Z*		−1.428	−2.270	−2.780		
	*p*		0.153	0.023	0.005		
MEF_50_	Control group	142	78.20 (63.40, 89.45)	85.10 (72.25, 93.08)	82.65 (69.93, 96.90)	13.443	0.001
	Experiment group	114	78.75 (66.15, 93.30)	88.40 (79.68, 101.40)	90.10 (82.20, 107.53)	33.719	0.000
	*Z*		−0.930	−2.617	−4.023		
	*p*		0.352	0.009	0.000		
MEF_25_	Control group	142	69.50 (53.68, 85.03)	74.30 (60.13, 88.15)	72.60 (58.83, 87.80)	9.894	0.007
	Experiment group	114	69.15 (52.15, 87.95)	80.15 (69.90, 93.28)	81.95 (68.85, 101.43)	37.903	0.000
	*Z*		−0.112	−2.839	−3.753		
	*p*		0.911	0.005	0.000		
MMEF	Control group	142	78.09 (63.30, 91.38)	84.00 (71.93, 96.50)	84.15 (70.15, 95.63)	12.236	0.002
	Experiment group	114	78.09 (63.33, 91.90)	88.65 (81.45, 102.03)	91.30 (80.10, 107.30)	35.860	0.000
	*Z*		−0.266	−2.930	−3.808		
	*p*		0.790	0.003	0.000		

### 3.2 Comparison of Airway Hyperresponsiveness Between the Two Groups of Patients

There was no significant difference in the baseline comparison of airway hyperresponsiveness between the two groups (*P* > 0.05). The difference in the comparison of airway hyperresponsiveness between the two groups was statistically significant after 6 months and 1 year of treatment (*P* < 0.05); and the difference in the comparison of the control group and the experiment group with the baseline was statistically significant after 6 months and after 1 year of treatment (*P* < 0.05). (Table 3).

**TABLE 3:** Comparison of airway hyperresponsiveness before and after treatment in the experiment and control groups.

Groups	*n*	Baseline (*n*, [%])	6 months (*n*, [%])	1 year (*n*, [%])	1 versus 2	1 versus 3	2 versus 3
Control group	142	141 (99.3)	133 (93.7)	124 (87.3)	0.010	0.000	0.069
Experiment group	114	112 (98.2)	96 (84.2)	86 (75.4)	0.000	0.000	0.099
*χ* ^2^		0.037	5.987	6.060			
*p*		0.848	0.014	0.014			

*Note:* 1, baseline, 2, 6 months of treatment, 3, 1 year of treatment.

### 3.3 Comparison of Inhaled Corticosteroid Dose Between the Two Groups of Patients

There was no significant difference in the baseline comparison of the pre‐treatment ICS inhalation dose between the two groups (*P* > 0.05), and the difference in the ICS inhalation dose between the two groups was statistically significant after 6 months and 1 year of treatment (*P* < 0.05). The difference in the comparison of the control group at all time points was statistically significant (*P* < 0.05) and the ICS inhalation dose was significantly lower after 1 year of treatment than that of baseline and after 6 months later (*P* < 0.05); and the difference in the comparison of the time points in the experiment group was statistically significant (*P* < 0.05), and the ICS inhalation dose was significantly lower than the baseline after 6 months and 1 year of treatment (*P* < 0.05) (Table 4).

**TABLE 4:** Comparison of inhaled corticosteroid dose before and after treatment in the experiment and control groups (ug).

Groups	The number of patient (*n*)	Baseline (M, [P₂₅, P₇₅])	6 months (M, [P₂₅, P₇₅])	1 year (M, [P₂₅, P₇₅])	*χ* ^2^	*p*
Control group	142	250 (200, 250)	250 (125, 250)	125 (125, 250)	59.785	0.000
Experiment group	114	250 (200, 250)	200 (125, 250)	125 (80, 160)	104.105	0.000
*Z*		−0.619	−2.732	−3.686		
*p*		0.536	0.006	0.000		

### 3.4 Comparison of Symptom Scores Between the Two Groups

Daytime symptoms: there was no significant difference in the comparison of daytime symptom scores between the two groups at baseline and after 6 months of treatment (*P* > 0.05). And after 1 year of treatment, the difference in the comparison of daytime symptom scores between the two groups was statistically significant (*P* < 0.05). The difference in the comparison of the control group at each time point was statistically significant (*P* < 0.05). The daytime symptom scores after 6 months and 1 year of treatment were significantly lower than baseline (*P* < 0.05), and daytime symptom scores after 1 year of treatment were significantly lower than after 6 months of treatment (*P* < 0.05). The difference between each time point comparisons in the experiment group was statistically significant (*P* < 0.05). And daytime symptom scores after 6 months and 1 year of treatment were significantly lower than baseline (*P* < 0.05), and daytime symptom scores after 1 year of treatment were significantly lower than after 6 months of treatment (*P* < 0.05) (Table 5).

**TABLE 5:** Comparison of daytime symptom scores before and after treatment in the experiment and control groups (point).

Groups	The number of patient (*n*)	Baseline (M, [P₂₅, P₇₅])	6 months (M, [P₂₅, P₇₅])	1 year (M, [P₂₅, P₇₅])	*χ* ^2^	*p*
Control group	142	2 (1.75, 2)	1 (0, 2)	1 (0, 1)	122.649	0.000
Experiment group	114	2 (1, 2)	1 (0, 1)	0 (0, 0.25)	160.465	0.000
*Z*		−0.396	−1.770	−4.947		
*p*		0.692	0.077	0.000		

Nocturnal symptoms: there was no significant difference in the baseline comparison of nocturnal symptom scores between the two groups (*P* > 0.05), and the difference in the comparison of nocturnal symptom scores between the two groups was statistically significant after 6 months and 1 year of treatment (*P* < 0.05). The difference in the comparison of the control group at each time point was statistically significant (*P* < 0.05), and nocturnal symptom scores were significantly lower than those of the baseline after 6 months and 1 year of treatment (*P* < 0.05). The difference in the comparison of time points in the experiment group was statistically significant (*P* < 0.05), and the nocturnal symptom scores were significantly lower than baseline after 6 months and 1 year of treatment (*P* < 0.05) (Table 6).

**TABLE 6:** Comparison of nocturnal symptom scores before and after treatment in the experiment and control groups (point).

Groups	The number of patient (*n*)	Baseline (M, [P₂₅, P₇₅])	6 months (M, [P₂₅, P₇₅])	1 year (M, [P₂₅, P₇₅])	*χ* ^2^	*p*
Control group	142	1 (1, 2)	1 (0, 1)	0 (0, 1)	77.076	0.000
Experiment group	114	1 (1, 2)	0 (0, 1)	0 (0, 0)	168.017	0.000
*Z*		−0.312	−5.228	−6.265		
*p*		0.755	0.000	0.000		

### 3.5 Comparison of the Efficacy of the Two Groups of Patients

The effective rate after 1 year of treatment in the control group was 86.6%, and the effective rate after 1 year of treatment in the experimental group was 94.7%. And the difference in total effective rate between the two groups after 1 year of treatment was statistically significant (*P* < 0.05) (Table 7).

**TABLE 7:** Comparison of therapeutic efficacy before and after treatment in the experiment and control groups.

Groups	The number of patient (*n*)	Marked Effect (*n*, [%])	Effective (*n*, [%])	Ineffective (*n*, [%])	Total effective rate (*n*, [%])
Control group	142	46 (32.4)	77 (54.2)	19 (13.4)	123 (86.6)
Experiment group	114	62 (54.4)	46 (40.5)	6 (5.3)	108 (94.7)
*χ* ^2^					4.728
*p*					0.030

## 4 Disscusion

### 4.1 Pathogenesis of Asthma and Mechanism on Action of SLIT

Bronchial asthma is a common chronic disease of the respiratory system in children, and with the changes in people's lifestyles and living environments, the prevalence of asthma is increasing year by year [6]. ICS and bronchodilators are commonly used medications for the treatment of asthma, but patients are prone to tolerate the use of these medications for a long period of time, and the incidence of adverse drug reactions is higher, making it difficult to achieve the expected results of treatment [7]. The pathogenesis of asthma is not completely clear, but it is currently believed that Th1/Th2 cytokine imbalance is the main pathogenesis of the disease. And the mechanism of airway inflammation in asthma patients is mainly due to the production of Th2‐associated cytokines, such as IL‐4, IL‐13, and IL‐5, secreted by Th2 cells and type 2 innate lymphocytes (ILC2s), which promotes the activation of mast cells, eosinophilic granulocyte infiltration and activation, and increases IgE production by B cells and induction of airway hyperresponsiveness [8, 9].

Specific immunotherapy as “allopathic treatment” was described by the World Health Organization (WHO) as “the only” treatment that can halt or reverse the natural course of allergic diseases. Studies have shown that sublingual specific immunotherapy aims to induce mucosal immunity against airway allergic reactions through allergen extracts, which promotes a temporary increase in the expression of high‐affinity IgE receptors on dendritic cells (DCs). This in turn leads to the development of allergen tolerance and a reduction in the local infiltration of basophils, mast cells, eosinophils, and their corresponding mediators. Additionally, regulatory dendritic cells (DCregs) induce regulatory T cells (Tregs), thereby promoting the shift of immune response from Th2 to Th1, restoring the Th1/Th2 balance to normal levels, and inducing the generation of regulatory B cell (Breg) subsets. IL‐10 derived from Bregs and Tregs contributes to the production of immunoglobulin IgG4 [10, 11].

### 4.2 SLIT on Asthma Symptom Control and Inhaled Glucocorticoid Dosing

It was shown that AIT leads to increased frequencies of IgA and IgG4 expressing Der p1 specific B cells, plasmoblasts and IL‐10^+^ Breg cells, which are significantly associated with clinical symptom improvement in AIT [12]. In this study, 1‐year observation of dust mite‐sensitized asthmatic children revealed that asthma symptoms and outcomes were significantly improved in both groups compared to pre‐treatment, more so in the experimental group, which suggests that SLIT reduces asthma symptoms and improves patients' quality of life. This study also found a statistically significant (*p* < 0.05) reduction in the dose of ICS used in the trial group compared to the control group after 1 year of treatment, which also potentially reduces the risk of adverse drug reactions. Pascal Demoly et al. found that the combination of AIT with ICS, through a review of 2 previous SCIT studies and 4 SLIT studies, is an effective and safe approach that it is possible to reduce the ICS dose while maintaining asthma control, with a more pronounced effect in patients with GINA class 3–4 (usually on moderate doses of ICS) [13].

### 4.3 Improvement of Asthmatic Lung Function and Airway Hyperresponsiveness by SLIT

Studies have shown that chronic inflammation and recurrent early and late allergic reactions in asthmatic airways may cause irreversible damage to the airways and increase airway sensitivity to a variety of nonallergic stimuli [14]. This is the main cause of reduced lung function in asthmatics. Mechanistically, ICS inhibits the production of chemotactic mediators, blocks the expression of adhesion molecules, and suppresses the survival of inflammatory cells in the airways [15, 16]. Thus ICS significantly improves lung function in asthma patients. AIT ultimately inhibits the differentiation and function of newly formed allergen‐specific Th2 cells by initiating a positive feedback loop of IL‐10 signaling and Treg‐mediated immunosuppression [17, 18]. This is also the rationale for the improvement of lung function in asthmatic patients by AIT. Makoto Hoshino et al. conducted a study on the effect of SLIT on airway inflammation and airway wall thickness in allergic asthma, and showed that ICS combined with SLIT significantly improved lung function and that this was independently correlated with a reduction in eosinophilic airway inflammation in response to improvement of airflow limitation by SLIT [19]. The present study demonstrated that SLIT combined with ICS treatment showed statistically significant improvement in the values of PEF, MMEF, MEF_75_, MEF_50_, and MEF_25_ indexes of lung function when compared to the group with ICS alone (*P* < 0.05), which is also in line with the previous report. The FEV1 index value showed significant improvement in the experimental group compared to the control group after 6 months of treatment (*p* < 0.05), and there was no significant difference between the two groups after 1 year of treatment (*p* > 0.05). The analysis is as follows: ① SLIT has a dose‐dependent effect. The onset of the effect requires at least 1–2 months, and the optimal onset of effect time in 3–6 months, so there is a difference in the comparison of the two groups after 6 months of treatment [20]; and ② ICS has a fast onset of action, and the optimal onset of action can be reached in about 3 weeks [21]. However, the ICS dose in the experimental group was significantly reduced compared with that in the control group at 1 year, so the lack of significant difference in the values of FEV1 indexes between the two groups at 1 year may be related to it and may further validate this hypothesis.

Antoon JM et al. found that immunotherapy was effective in reducing airway eosinophilia and hyperreactivity by establishing a mouse model of allergic asthma. Furthermore, this effect of immunotherapy was associated with a decrease in IL‐4 production by lymphocytes and a shift in serum antibody isotype [22]. This study showed that the positive rate of airway hyperreactivity was 75.4% and 87.3% after 1 year of treatment in the test group and control group patients, respectively, and the difference was statistically significant (*P* < 0.05), which indicated that SLIT could improve airway hyperreactivity in patients with bronchial asthma, reduce the risk of acute asthma exacerbation, and improve the safety of asthma treatment.

### 4.4 Recent Advances in SLIT and Shortcomings of This Study

It has been suggested that there is a global effect of AIT that allows T and B regulatory cells to inhibit effector cell responses to other allergens, thus preventing the development of new allergic sensitization [23, 24]. Maurizio Marogana et al. conducted a randomized controlled study on the prophylactic effect of SLIT in allergic rhinitis, and the results showed that SLIT can reduce new sensitization in fewer patients [25]. However currently there is only a low level of evidence to support the preventive effect of AIT on new‐onset sensitization, and the long‐term effects of discontinuing AIT are not supported by studies, so further clinical studies should be conducted to investigate this hypothesis.

However, the shortcomings of this study are that the observation time is relatively short, and it is impossible to verify the long‐term effectiveness and allergen immune tolerance of SLIT. Moreover, the authors noted that the values of lung function indexes in this study and similar studies were mostly at normal levels, and the comparison between the expeirment group and the control group only rested on the change of the values, which could not clearly analyze whether SLIT could improve small airway dysfunction. Because this study is a retrospective study, the compliance of asthma patients can only be judged by the number of follow‐up visits, so the true compliance of patients cannot be clarified.

## 5 Conclusion

In conclusion, this study demonstrates that sublingual specific immunotherapy combined with inhaled corticosteroid therapy has a positive therapeutic effect on asthma patients, which can reduce the dose of medication used by asthma patients and improve symptoms. Meanwhile, this study also provides further evidence for the role of SLIT in asthma treatment, but the results of different studies may vary because of different inclusion criteria, treatment duration, and sample size. Therefore, further multicenter, large‐sample‐size, and long‐term studies are needed to verify the long‐term efficacy and safety of SLIT in asthma treatment.

## Author Contributions

**Yinming Song:** methodology (lead), investigation (lead), formal analysis (lead), writing – original draft (lead). **Daiyin Tian:** writing – review and editing. **Sha Liu:** writing – review and editing. **Fangjun Liu:** writing – review and editing. **Jingyue Liu:** writing – review and editing. **Ying Li:** writing – review and editing. **Li Yan:** conceptualization (lead), supervision (lead), writing – review and editing (lead).

## Conflicts of Interest

The authors declare no conflicts of interest.

## Ethics Statement

This study was reviewed by the Medical Ethics Committee of Children's Hospital of Chongqing Medical University (approval number: 2023, 505).

## Data Availability

The authors have nothing to report.
